# To explore the prognostic characteristics of colon cancer based on tertiary lymphoid structure-related genes and reveal the characteristics of tumor microenvironment and drug prediction

**DOI:** 10.1038/s41598-024-64308-w

**Published:** 2024-06-12

**Authors:** Zhanmei Wang, Dongguang Niu

**Affiliations:** 1 Department of Oncology, Qilu Hospital of Shandong University, Qingdao, 266000 China; 2 Department of Gastrointestinal Surgery, Affiliated Hospital of Qingdao University, Qingdao City, 266000 Shandong Province China

**Keywords:** Colon adenocarcinoma, Tertiary lymphoid structures, Tumor microenvironment, Drug sensitivity, Prognosis, Immune, Gastrointestinal cancer, Cancer, Drug discovery

## Abstract

In order to construct a prognostic evaluation model of TLS features in COAD and better realize personalized precision medicine in COAD. Colon adenocarcinoma (COAD) is a common malignant tumor of the digestive system. At present, there is no effective prognostic marker to predict the prognosis of patients. Tertiary lymphoid structure (TLS) affects cancer progression by regulating immune microenvironment. Mining COAD biomarkers based on TLS-related genes helps to improve the prognosis of patients. In order to construct a prognostic evaluation model of TLS features in COAD and better realize personalized precision medicine in COAD. The mRNA expression data and clinical information of COAD and adjacent tissues were downloaded from the Cancer Genome Atlas database. The differentially expressed TLS-related genes of COAD relative to adjacent tissues were obtained by differential analysis. TLS gene co-expression analysis was used to mine genes highly related to TLS, and the intersection of the two was used to obtain candidate genes. Univariate, LASSO, and multivariate Cox regression analysis were performed on candidate genes to screen prognostic markers to construct a risk assessment model. The differences of immune characteristics were evaluated by ESTIMATE, ssGSEA and CIBERSORT in high and low risk groups of prognostic model. The difference of genomic mutation between groups was evaluated by tumor mutation burden score. Screening small molecule drugs through the GDSC library. Finally, a nomogram was drawn to evaluate the clinical value of the prognostic model. Seven TLS-related genes ADAM8, SLC6A1, PAXX, RIMKLB, PTH1R, CD1B, and MMP10 were screened to construct a prognostic model. Survival analysis showed that patients in the high-risk group had significantly lower overall survival rates. Immune microenvironment analysis showed that patients in the high-risk group had higher immune indicators, indicating higher immunity. The genomic mutation patterns of the high-risk and low-risk groups were significantly different, especially the KRAS mutation frequency was significantly higher in the high-risk group. Drug sensitivity analysis showed that the low-risk group was more sensitive to Erlotinib, Savolitinib and VE _ 822, which may be used as a potential drug for COAD treatment. Finally, the nomogram constructed by pathological features combined with RiskScore can accurately evaluate the prognosis of COAD patients. This study constructed and verified a TLS model that can predict COAD. More importantly, it provides a reference standard for guiding the prognosis and immunotherapy of COAD patients.

## Introduction

Colon adenocarcinoma (COAD) is a common malignant tumor, accounting for the third highest incidence of malignant tumors in the world. Due to the lack of clear early signs of disease, the prognosis of COAD patients is not ideal, and its mortality rate is still the second highest in the world^1–3^. The development of immunotherapy has improved the prognosis of patients with COAD. At present, immune checkpoint inhibitors have shown efficacy in patients with advanced COAD, and their efficacy is considered to be related to tumor mutation burden (TMB)^4,5^. However, there are still some COAD patients with high mutation load who cannot benefit from immunotherapy. Therefore, TMB alone cannot evaluate the efficacy and prognosis of immunotherapy for all types of COAD^6^. A retrospective study of colorectal cancer tissues showed that the response to immunotherapy in patients with high mutation load was highly correlated with the heterogeneity of tumor microenvironment (TME). In particular, the infiltration level of CD8 + T cells and CD74 + macrophages in TME was directly related to the patient’s long-term benefit from immunotherapy^7,8^. Therefore, the current research urgently needs to explore biomarkers that can effectively evaluate the immune characteristics of TMB and TME in COAD patients, and provide guidance for the improvement of COAD immunotherapy.

Tertiary lymphoid structures (TLS) are immune cell aggregates formed by tumors under chronic inflammatory conditions^9^. The aggregates are ectopic lymphoid structures composed mainly of B cells, T cells and dendritic cells^10^. Studies have shown that the infiltration of TLS is closely related to the prognosis of cancer patients and the efficacy of immunotherapy^10,11^. On the one hand, the formation of TLS is associated with better clinical and prognostic outcomes in most cases. For example, studies have found that TLS can improve the prognosis of patients with metastatic melanoma by regulating T cell phenotype, which is reflected in the lack of TLS structure in patients with T cells in cancer tissues with dysfunctional molecular phenotypes^12^. Similarly, the spatial distribution and abundance of TLS in intrahepatic cholangiocarcinoma are significantly related to the prognosis of patients, especially the progress of regulating the distribution of follicular helper T cells and regulatory T cells in tissues^13^. In colorectal cancer-related studies, high TLS density is considered to be a favorable prognostic factor for patients with non-metastatic colorectal cancer, which exerts anti-tumor immune activity mainly through the infiltration of CD20 + B cell, CD45RO + lymphocytes, CD4 + T cell and CD8 + T cell^14^. On the other hand, TLS has an important contribution to the anti-tumor immune response of TME and can be used as an evaluation index for immunotherapy. In terms of immune cell infiltration regulation, CD20 + B cells are localized in TLS of various types of tumors and co-localized with CD4 + T cells, CD8 + T cells, and CD21 + follicular dendritic cells. The infiltration of these cells can be used as an important indicator of adjuvant immunotherapy^15^. From the specific mechanism, immune cells in TLS play a key role in the response to PD-L1 / PD-1 by secreting antibodies and cytokines^16^. Among them, dendritic cells regulate the process of antigen presentation and regulate the function of T cells, while B cells have dual mechanisms of tumor promotion and anti-tumor, and their related functions depend on the mature state of TLS, and the cytokines released by B cells and dendritic cells in TLS can synergistically activate CD8 + T cells^17^. TLS is of great significance both in evaluating the prognosis of cancer patients and as a clinical therapeutic target. Therefore, mining potential therapeutic targets of COAD characterized by TLS will be the key entry point of this study.

In this study, seven characteristic genes were screened based on the co-expression network of TCGA-COAD differentially expressed genes and TLS-related genes to construct a prognostic model. The samples were divided into high-risk and low-risk groups by risk score, and the differences in overall survival rate, immune characteristics, genomic mutation frequency and drug sensitivity between groups were evaluated, which verified the accuracy of the model in evaluating cancer-related characteristics. Finally, a nomogram that can accurately evaluate the prognosis of COAD patients was constructed based on pathological features and RiskScore. The prognostic biomarkers and their characteristics analyzed in this study are expected to provide guidance for the early diagnosis and clinical treatment of COAD.

## Materials and methods

### Source of data analysis

The mRNA expression data of colon cancer (COAD) TCGA-COAD (normal: 41, tumor: 480), SNP data and clinical information were downloaded from the TCGA database (https://portal.gdc.cancer.gov/) as the training set. GSE17536 was downloaded from the GEO database (https://www.ncbi.nlm.nih.gov/gds) as a validation set. In the follow-up study, 39 TLS-related genes were from the existing reports^18^.

### Construction of prognostic model based on TLS-related genes

R package ‘edgeR’ was used to analyze the difference of TCGA-COAD (| logFC | > 1, FDR < 0.05), and the differentially expressed genes (DEGs) of COAD samples relative to adjacent samples were obtained. The co-expression analysis of 39 TLS-related genes was performed by Cytoscape (https://cytoscape.org/)) (r > 0.5, *p* < 0.05), and the genes highly related to TLS were screened. The intersection of these genes with DEGs was used as a candidate gene for subsequent analysis.

Univariate Cox regression analysis of candidate genes was performed using the R package ‘survival’ to screen genes significantly associated with prognosis (*P* < 0.01). In order to prevent over-fitting of the model, R package ‘glmnet’ was used to perform Lasso-Cox regression analysis on the genes screened by univariate Cox regression analysis to obtain candidate feature genes. R package ‘survival’ was used to further screen the candidate characteristic genes by two-way stepwise regression method, and a multivariate Cox regression model was constructed for the genes significantly associated with prognosis, and the forest map was drawn by R package ‘survminer’.

### Prognostic model validation

According to the median value of the model risk score, the samples were divided into high and low risk groups, and the R package ‘pheatmap’ was used to draw the model characteristic gene heat map, as well as the risk score distribution map and the survival status distribution map. The R package ‘FactoMineR’ and ‘factoextra’ were used to perform PCA dimensionality reduction analysis on the model feature genes according to the high and low risk groups to verify the grouping effect based on the model feature genes. Kaplan–Meier survival analysis was performed on the high and low risk groups using the R package ‘survival’, and the ROC curve was drawn using the R package ‘timeROC’ to obtain the AUC values of the 1-, 3-, and 5-year overall survival (OS). The above steps are verified in GSE17536.

### Analysis of immune microenvironment in high and low risk groups

The R package ‘estimate’ was used to evaluate the tumor microenvironment of each sample in high and low risk, and the immune score, matrix score, ESTIMATE score and tumor purity of each sample were calculated. R package ‘GSVA’ was used for ssGSEA analysis to visualize the differential enrichment of immune-related cells and functions in high and low risk groups. The ‘CIBERSORT’ package was used to analyze the differences of 22 immune cell infiltration levels in the high-and low-risk groups.

### Analysis of mutation characteristics in high and low risk groups

R package ‘maftools’ was used to visualize the mutation sites of high-frequency mutant genes in high-and low-risk groups, and to explore the co-mutation and mutual exclusion between them.

### Analysis of small molecule drug sensitivity in high and low risk groups

Cancer drug sensitivity genomics (GDSC) is a public data set that contains information on cancer cell drug sensitivity and drug response molecular markers. The GDSC2 gene expression profile and corresponding drug response information were downloaded using the R package ‘oncoPredict’to generate a ridge regression model that can be applied to COAD transcriptome data, and then a sensitivity score was generated to predict the maximum half-inhibitory concentration (IC50) of the drug acting on the patient. Based on the t-test analysis of IC50 between high and low risk groups, and using the ‘ggpubr’ package to draw a violin map, the potential therapeutic drugs for COAD patients were explored.

### Prognostic risk model independence verification and nomogram construction

In the training set, univariate Cox regression analysis was performed using the R package ‘survival’ in combination with clinical features and risk scores, and forest plots were drawn using the R package ‘forestplot’. Multivariate Cox regression analysis was performed using the R package ‘survival’, and the forest plot was drawn using the R package ‘forestplot’ to determine whether the risk score could be used as an independent prognostic factor. Combined with clinical features and risk scores, the R package ‘rms’ was used to construct a nomogram, and the 1-, 3-, and 5-year correction curves were drawn to evaluate the accuracy of the nomogram in predicting the prognosis of patients.

### Statistical analysis

All statistical data were analyzed using the R language (version 3.6.0) and wcoxon sum tests were used to calculate differences between two groups of continuous variables. Correlations were calculated using the ssaman method. The logrank test was used to compare the differences in survival time between patients in each group. In all analyses, *p* < 0.05 was considered statistically significant.

## Results

### Construction and validation of COAD prognostic model based on TLS-related genes

**Figure 1:**
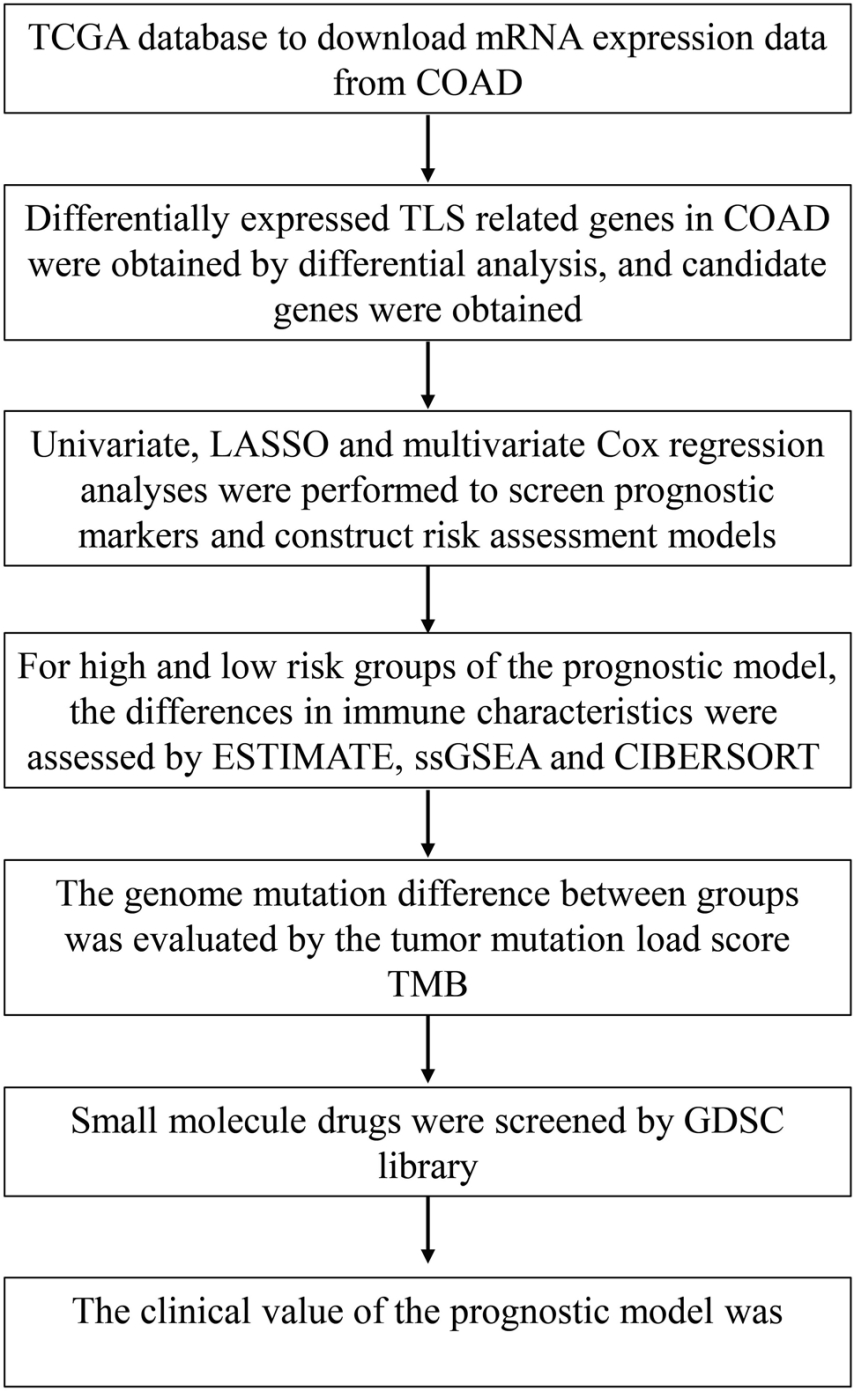
Flowchart of this study.

**Figure 2:**
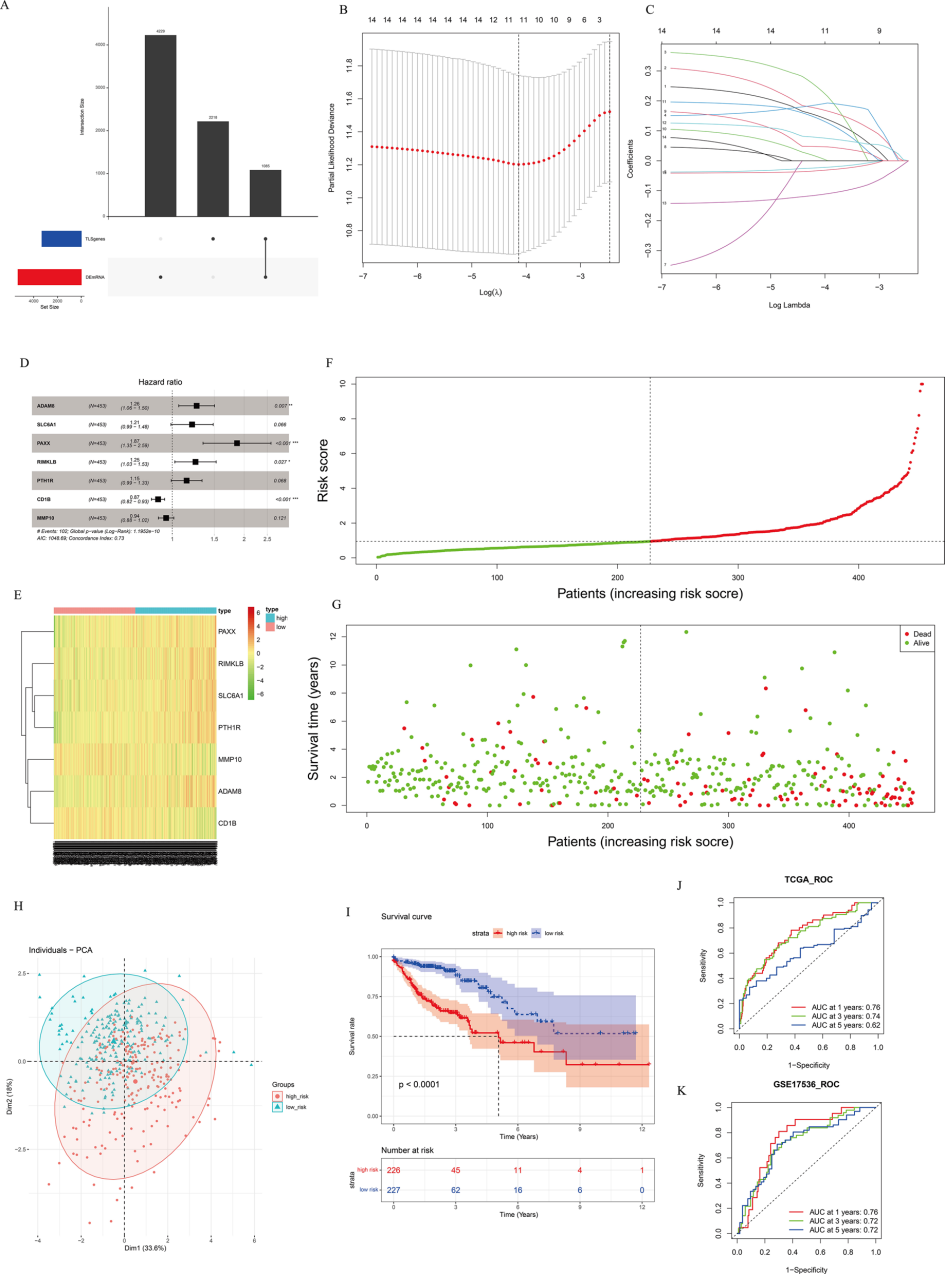
Construction and verification of colon cancer prognosis model. (**A**) Based on the intersection of differentially expressed genes and TLS highly related genes, candidate genes were screened. (**B**) Screening the best penalty parameter (λ) of lasso cox regression model. (**C**) lasso cox regression analysis of gene coefficient spectrum. (**D**) Multi-factor cox regression analysis forest map. (**E**) 7 characteristic gene expression thermograms of prognostic models between high and low risk groups. (**F**) RiskScore distribution map of patients. (**G**) Survival state distribution map. (**H**) PCA distribution map of the characteristic genes of the high and low risk group model. (**I**) Survival analysis of high and low risk group samples. (**J**) ROC curve of TCGA training set samples. (**K**) ROC curve of validation set samples.

The flow chart of this study is shown in Fig. 1. In this study, a total of 1085 TLS-related differentially expressed genes (DEGs) were obtained from the intersection of the genes obtained by differential analysis and the results of co-expression analysis (Fig. 2A). Univariate Cox regression analysis combined with Lasso-Cox regression analysis of these genes resulted in 11 candidate characteristic genes (Fig. 2B,C). Multivariate Cox regression analysis was performed on 11 candidate characteristic genes, and finally 7 characteristic genes related to TLS and prognosis were obtained (Fig. 2D).A prognostic model was constructed for the characteristic genes according to the following formula :$$\begin{aligned} {\text{RiskScore}} & = 0.2348 \times {\text{ADAM}}8 + 0.1909 \times {\text{SLC}}6{\text{A}}1 + 0.6241 \times {\text{PAXX}} \\ & \quad + 0.2238 \times {\text{RIMKLB}} + 0.1385 \times {\text{PTH}}1{\text{R}}{-}0.1366 \times {\text{CD}}1{\text{B}}{-}0.0589 \times {\text{MMP}}10 \\ \end{aligned}$$.

The characteristic gene expression heat map of the model showed that PAXX, RIMKLB, SLC6A1, PTH1R, ADAM8 genes were highly expressed in the high-risk group, while MMP10 and CD1B were lowly expressed in the high-risk group (Fig. 2E). The RiskScore distribution map shows that RiskScore is higher in the high-risk group (Fig. 2F). At the same time, the survival status distribution map shows that the number of patient deaths gradually increases with the increase of RiskScore (Fig. 2G). PCA analysis showed that 7 characteristic genes could well distinguish high and low risk group samples (Fig. 2H). The results of survival analysis showed that the overall survival rate of patients in the high-risk group was lower and showed a worse prognosis level (Fig. 2I). ROC analysis showed that the AUC values of the TCGA-COAD set for predicting the 1-, 3-, and 5-year prognostic survival rates of patients were 0.76,0.74, and 0.62, respectively (Fig. 2J). The AUC values of the validation set GSE17536 for predicting the 1-, 3-, and 5-year prognostic survival rates of patients were 0.76, 0.72, and 0.72, respectively (Fig. 2K). The above results indicate that the 7 gene model has a good predictive effect on the prognosis of COAD patients.

### Analysis of immune components in high and low risk groups

**Figure 3:**
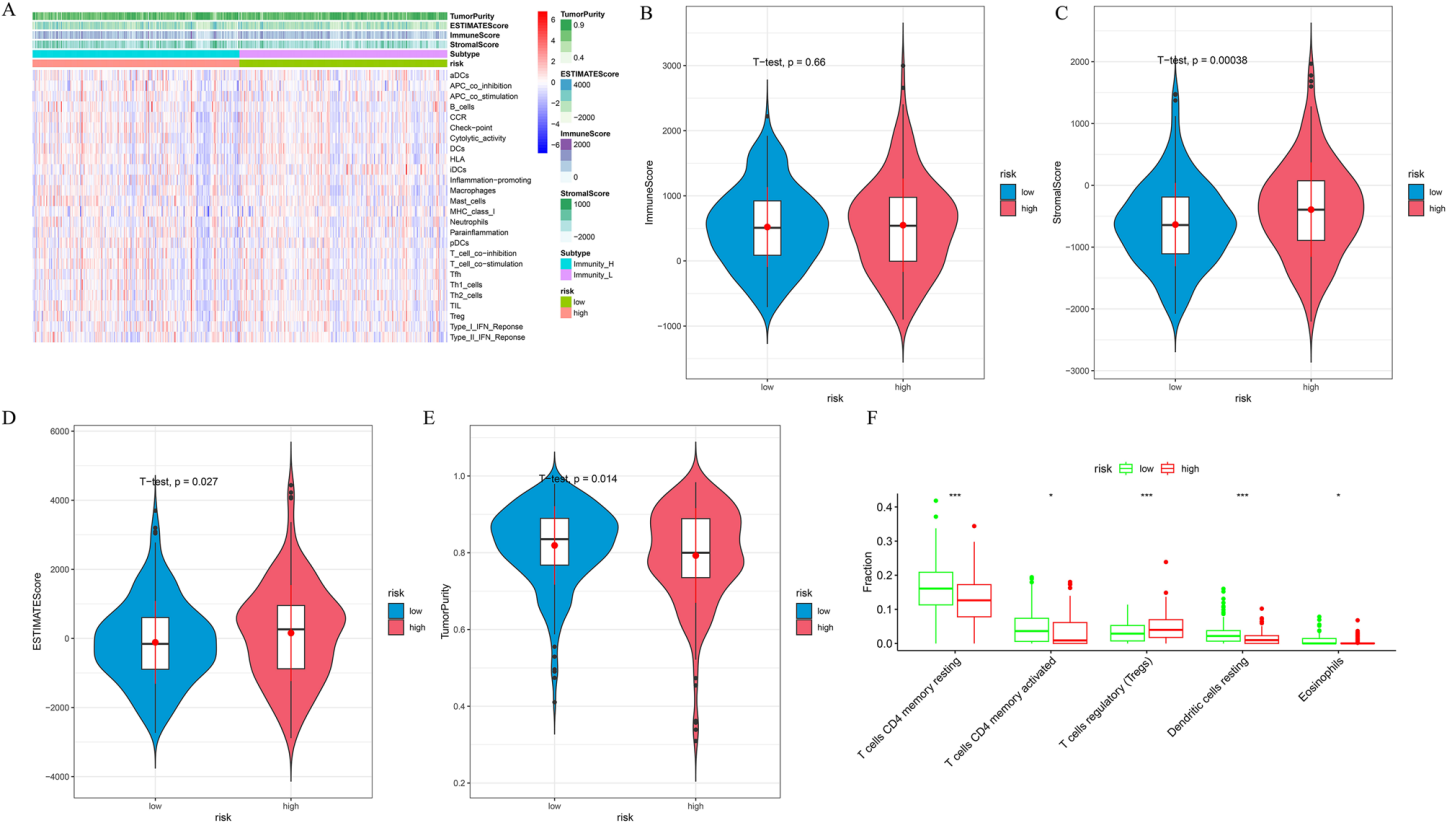
Prognostic model to evaluate the reliability of COAD immune components. (**A**) Enrichment difference heat map of immune components and immune cells between high and low risk groups. The immune score (**B**), matrix score (**C**), ESTIMATE (**D**) score and tumor purity (**E**) were differentially expressed in the high and low risk groups. (**F**) CIBERSORT visualized the infiltration difference of immune cells between high and low risk groups.

In order to explore the relationship between the model RiskScore and the regulation of immune infiltration microenvironment, this study evaluated the differences in immune cell scores and immune component scores between high and low risk groups, and found that the levels of these immune indicators in the high-risk group were higher than those in the low-risk group (Fig. 3A). At the same time, the matrix score and ESTIMATEScore in the high-risk group were significantly higher than those in the low-risk group, while the tumor purity was significantly lower than that in the low-risk group (Fig. 3B,E). Perhaps stromal cells play an important role in the high-risk group, which remains to be elucidated in subsequent studies. CIBERSORT analysis showed that Tregs had significantly higher infiltration levels in the high-risk group, while resting CD4 + T memory cells and dendritic cells had significantly lower infiltration levels in the high-risk group (Fig. 3F). These results indicate the differences in immune characteristics between high-and low-risk groups, and the lower level of immune component infiltration in the high-risk group also suggests worse anti-tumor immune activity.

### Differences in mutation characteristics between high and low risk groups

**Figure 4:**
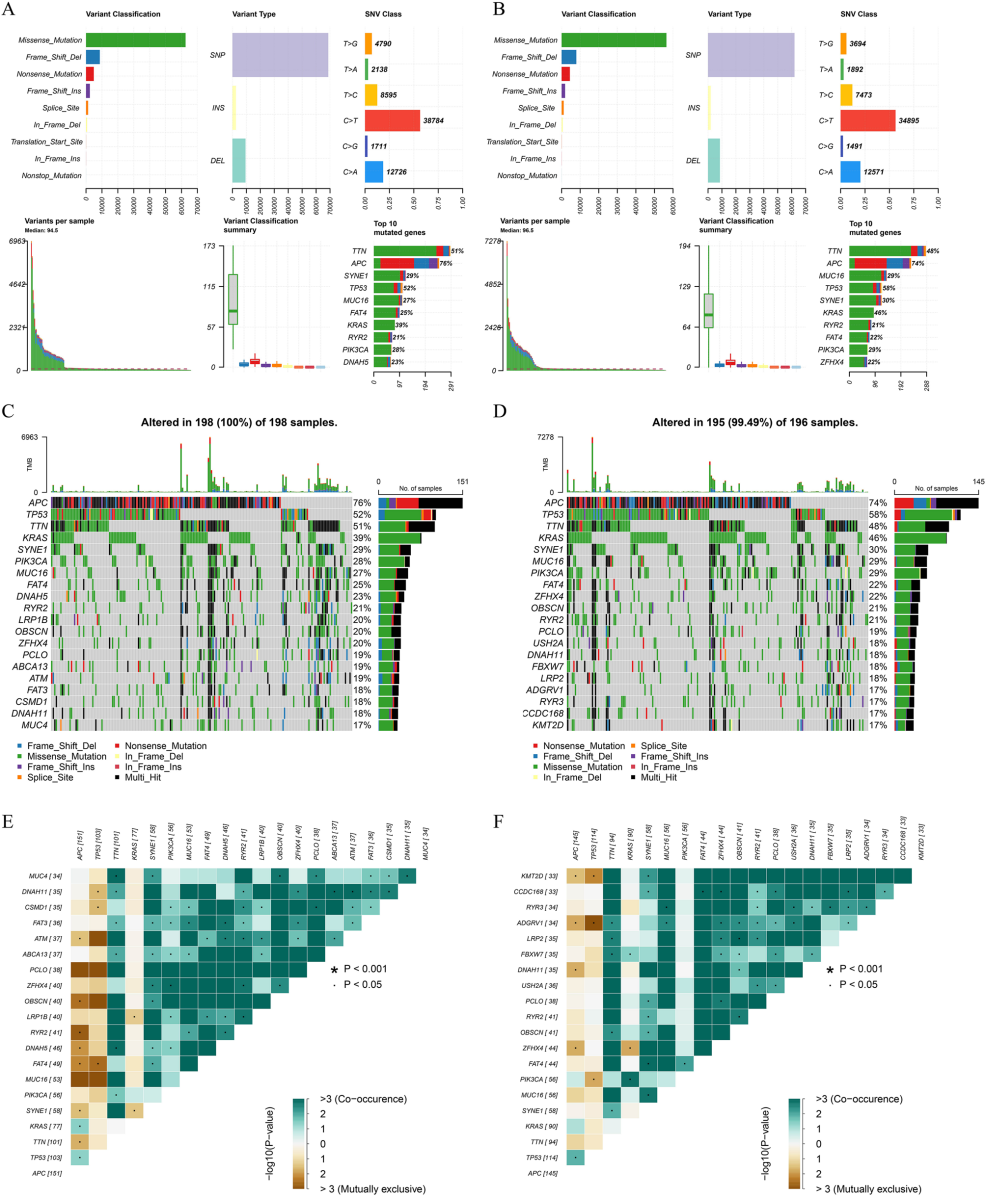
Analysis of genomic mutation frequency in high and low risk groups. (**A**) Statistics of high-frequency mutation genes, mutation sites, and mutation types in the low-risk group. (**B**) Statistical map of high frequency mutation genes, mutation sites and mutation types in high risk group. (**C**) The waterfall diagram of the mutation frequency top20 gene in the low-risk group. (**D**) Waterfall map of top20 gene mutation frequency in high-risk group. (**E**) Co-mutation and mutually exclusive mutation map of top20 gene in low-risk group. (**F**) The co-mutation and mutually exclusive mutation of the top20 gene in the high-risk group.

The TMB score of each sample was calculated by the number of mutations per million bases. The top 20 genes of mutation frequency in the high and low risk groups were analyzed. There were differences in the top 20 mutation genes between the high and low risk groups, and the mutation frequency was also different. It is worth noting that the frequency of KRAS mutations in the high-risk group was significantly higher than that in the low-risk group (Fig. 4A–D). The gene co-mutation and mutually exclusive mutation map showed that the proportion of co-mutation and mutually exclusive mutation in the low-risk group was larger than that in the high-risk group (Fig. 4E,F). These results indicate the heterogeneity of gene mutation patterns between high and low risk groups, which may be one of the factors leading to differential prognosis between samples.

### Sensitivity analysis of small molecule drugs in high and low risk groups

In order to clarify the differences in the sensitivity of chemotherapy drugs between the high and low risk groups of the prognostic model, this study explored the differences in the IC50 values of various chemotherapy drugs acting on the samples between the high and low risk groups. The results showed that the three chemotherapeutic drugs Erlotinib, Savolitinib, and VE _ 822 had significantly higher IC50 values in the high-risk group (Fig. 4A–C). This also represents a higher resistance to three anticancer drugs in the high-risk group.

### Prognostic model independence verification and clinical value evaluation

**Figure 5:**
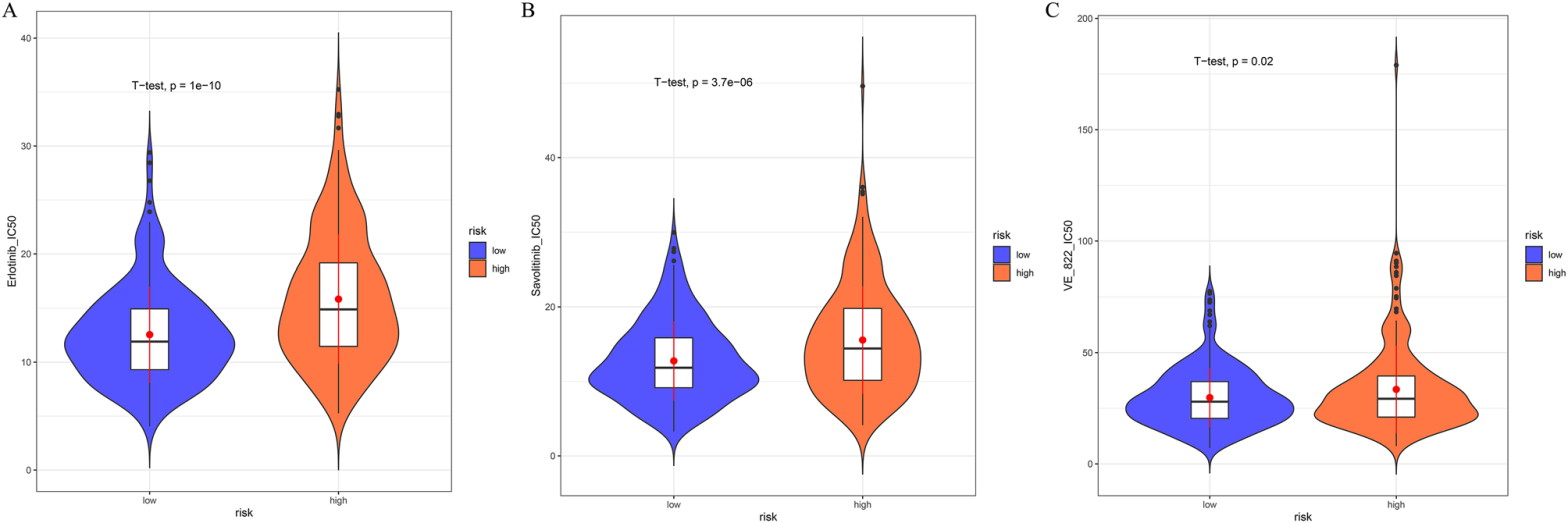
Difference evaluation of small molecule drug sensitivity of samples. (**A**) Violin plot showed that the IC50 of Erlotinib in the treatment of CC was different in the high and low risk groups. A violin plot (**B**) showed the difference in IC50 of CC treated with Savolitinib between the high-and low-risk groups. (**C**) Violin plot showed the difference of IC50 of drug VE _ 822 treated CC in high and low risk groups.

**Figure.6:**
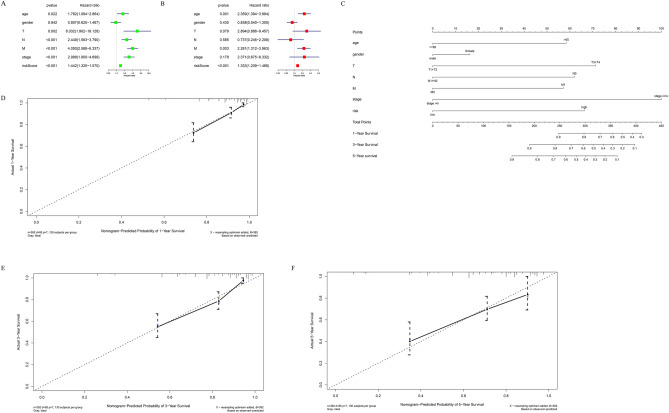
Column diagram construction and evaluation. (**A**,**B**): univariate Cox regression analysis (**A**) and multivariate Cox regression analysis (**B**) forest plot of clinical traits, risk score and OS. (**C**) Nomogram for predicting 1-, 3-, and 5-year OS in CC patients by combining 7-feature gene risk score and other clinical factors. (**D**–**F**): The nomogram was used to predict the correction curve of 1-year (**D**), 3-year (**E**), and 5-year (**F**) survival rates.

In the TCGA training set, this study performed univariate Cox regression analysis on the risk score combined with clinical information. The results showed that T, N, M, stage, and RiskScore were significant for the prognosis of patients (Fig. 6A). The results of multivariate Cox regression analysis showed the same conclusion (Fig. 5B). This shows that the prognostic model RiskScore constructed in this study can be used as an independent prognostic factor. Then, this study used clinical information combined with risk status to construct a nomogram, and constructed a calibration curve verification model to evaluate the good performance of patient’s 1-, 3-, and 5-year prognostic survival rates (Fig. 6C–F).

## Discussion

For cancer patients, the formation of tertiary lymphoid structure (TLS), as a local infiltrating microenvironment, affects disease progression through the activation of immune cells, and is often used as a marker to predict a good prognosis of cancer. By analyzing the genomic characteristics associated with TLS formation in pan-cancer and its interaction with the tumor immune microenvironment, TLS has been identified as a prognostic predictor of various cancers, including COAD^19^. Our study aims to construct a reliable prognostic assessment model by mining biomarkers in COAD patients, which is important for achieving personalized treatment and improving patient survival. Although studies have been conducted to explore multiple biomarkers of COAD, there is still a relative lack of systematic studies based on genes related to TLS. TLS plays a key role in regulating immune responses in the tumor microenvironment, and thus we believe that studies based on TLS-associated genes will fill this gap. We chose to use well-established bioinformatics tools and methods (e.g., differential expression analysis, co-expression network analysis, and Cox regression modeling) because the validity and reliability of these methods have been validated in multiple studies. The use of these tools ensures the robustness of our analysis and the accuracy of our results. Our work analyzes TLS-associated genes by integrating multiple bioinformatics methods, which not only increases the depth of interpretation of the available data, but also improves the likelihood of discovering potential biomarkers. The effectiveness of the response provides a basis for the diagnosis and treatment of COAD diseases.

Among the seven genes involved in the construction of the prognostic model, PAXX, RIMKLB, SLC6A1, PTH1R, and ADAM8 genes were used as prognostic risk factors, while MMP10 and CD1B were used as prognostic protective factors. Studies have shown that PAXX can be used as an independent prognostic factor for colon cancer patients, and its expression is significantly up-regulated in colon cancer tissues. Its high expression is related to the down-regulation of cytotoxic lymphocyte infiltration and the activation of several carcinogenic pathways, especially the oxidative stress response pathway^20^. This is consistent with the trend of this study to predict the gene as a poor prognostic factor. RIMKLB can be associated with a worse prognosis of COAD patients by affecting the activation of immune cells. The expression level of RIMKLB is positively correlated with the expression levels of PD-1, PD-L1, CTLA4 and other proteins in TME, and the high expression of these proteins is usually not conducive to the efficacy of immunotherapy^21–23^. This study further revealed that RiskScore was negatively correlated with immune effector cells such as CD4 + T cells, further confirming that the gene is not conducive to the prognosis of patients through immunosuppressive response. SLC6A1 is usually used as an important mediator of neurodevelopmental disorders. There are many studies in glioma, which is associated with the poor prognosis of this type of cancer^22,23^. In recent years, a study has excavated this gene as a marker of mitophagy-related genes in COAD, and predicted that patients with high expression of SLC6A1 were more likely to have immune escape response by means of bioinformatics^24^. Therefore, the expression of this gene may also be an adverse prognostic factor through immunosuppressive response. PTH1 R induces the migration phenotype of cancer cells such as breast cancer and prostate cancer by encoding parathyroid hormone and inducing the disorder of cell metabolic pathways such as calcium metabolism and glucose metabolism, which is usually used as an unfavorable factor for patients^25,26^. The high expression of ADAM8 is closely related to cancer cell migration and chemotherapy drugs. The protein encoded by this gene can not only mediate the hydrolysis of extracellular matrix proteins to release angiogenic factors to promote cancer progression, but also cooperate with integrins to activate ERK, AKT and other pathways to promote chemotherapy resistance^27^. The research trend of these genes as adverse prognostic factors of patients is consistent with the conclusion of this paper. At the same time, MMP10 and CD1 B as prognostic protective factors have also been confirmed in related studies^28,29^. MMP10 improves cancer progression and promotes the sensitivity of patients to radiotherapy through DNA damage repair^30^. CD1B, a member of the glycoprotein family, plays an important role in T cell antigen presentation. Therefore, in T cell adoptive therapy, the presence of this protein has effectively exerted the therapeutic effect of adjuvant anti-tumor immunity^31,32^. This provides a reference for this article, that is, the expression of this gene may improve the prognosis of patients through the activation of T cells in TME, which is also consistent with the conclusion that there is a significant difference in T cell infiltration level between the high and low Riskscore groups in this study. These genes were identified as being associated with TLS in this study. In previous research, the presence of TLS across a broad spectrum of cancers has often been viewed as a marker of favorable prognosis. For instance, in melanoma and breast cancer, a high density of TLS has been strongly linked to improved patient survival. This aligns with our observations of TLS-associated genes in colorectal cancer, suggesting that TLS may play a beneficial immunomodulatory role in various cancers^12^. However, our study also highlights differences from those reported in TLS studies of other cancer types. Specifically, in colorectal cancer, TLS-associated genes such as ADAM8 and SLC6A1 are linked to disease progression and immune escape mechanisms, findings that are relatively uncommon in the literature for other cancer types^24,33^. This may reflect the unique tumor microenvironment and immune landscape specific to colorectal cancer. Our findings emphasize the importance of exploring TLS function across different tumor types and suggest that even with similar TLS densities, TLS may influence disease progression and therapeutic responses through distinct biological mechanisms in different cancers. These TLS-associated genes collectively affect colorectal cancer progression and patient prognosis by regulating multiple aspects of the tumor microenvironment, including immune response, cell migration, intercellular communication, and cell metabolism. Their expression and mechanisms of action not only provide new insights into the biology of COAD, but also provide a potential basis for the development of therapeutic strategies against these targets. In summary, the genes related to the prognosis of COAD in this study are generally associated with the malignant progression or immune regulation of tumor cells, and can be used as biomarkers for predicting the prognosis of patients and potential targets for immunotherapy.

Subsequently, this study established differential enrichment of immune characteristics in high and low RiskScore samples, and found that Tregs had significantly higher infiltration levels in the high-risk group, while resting CD4 + T memory cells and dendritic cells had significantly lower infiltration levels in the high-risk group. Regulatory T cells (Tregs) play an immunosuppressive role in TME of most solid tumors. Similarly, in TLS, the high level of Tregs infiltration inhibits the proliferation and cytokines of cytotoxic T lymphocytes by inhibiting the related antibodies targeting CTLA4, and down-regulates its anti-tumor activity, resulting in poor prognosis of patients^34–36^. CD4 + T cells and dendritic cells are known to exert anti-tumor activity. CD4 + T cells mediate anti-tumor immunity by assisting the production of CD8 + T effector cells, activating innate immune response and anti-tumor angiogenesis, and are often used as candidates for cancer adoptive therapy^37^. Dendritic cells play a key role in mediating cytotoxicity and inhibiting tumor angiogenesis. These factors are closely related to the improvement of prognosis in patients with advanced colon cancer. In view of the outstanding effect of dendritic cells on anti-tumor immunity, the related drugs based on dendritic cell adoptive therapy have been approved by FDA^38^. Therefore, in this study, the high-risk group samples showed immunosuppressive activity, while the low-risk group showed anti-tumor activity. The difference in immune characteristics between samples verifies the rationality of predicting tumor immune pattern based on TLS-related gene prognostic model, which will also provide a basis for the application of this model in the clinical treatment of COAD.

In order to explore the relationship between the prognostic model and the drug sensitivity of COAD, this study elucidated the correlation between the RiskScore of the model and the IC50 of anticancer drugs. We revealed the resistance of the high-risk group to chemotherapy drugs Erlotinib, Savolitinib, and VE _ 822. A cell experiment verified that Erlotinib can inhibit CXCL8-induced colon cancer cell metastasis, ERK and AKT pathways^39^. Savolitinib exerts an anti-tumor effect by inhibiting mutations in the epidermal growth factor receptor. It is usually combined with Osimertinib to treat cancer types that are highly resistant due to genomic mutations^40^. VE_822 has the function of DNA damage repair and inhibits the proliferation and migration of cancer cells by inducing apoptosis. It has considerable clinical efficacy in patients with ovarian cancer, lung adenocarcinoma and head and neck squamous cell carcinoma^41–44^. It can be seen that the prognostic model constructed by mining TLS-related genes in this study can be used to evaluate the drug response of COAD patients and provide reference for clinical medication of COAD.

The presence of TLS is closely related to the activity and distribution of immune cells in tumors. Since TLS can promote effective immune responses, its changes during the generation of CTCs may affect the immune system's recognition and clearance of tumor cells that break away from the tumor and enter the blood circulation. Therefore, the formation of CTCs may be affected to some extent by the TLS status^45,46^. In tumor microenvironments with abnormal TLS function or expression of specific TLS-related genes, the characteristics of CTCs may vary, including their ability to survive, metastatic potential, and response to therapy^47–49^. Therefore, monitoring these changes through CTCs technology can provide important information about the status of TLS and its regulatory mechanisms. By combining the detection of CTCs with the study of TLS, tumor response to treatment can be more accurately assessed, especially in immunotherapy and radiotherapy. For example, if treatment leads to changes in TLS properties, this may be reflected in changes in the number and properties of CTCs, thereby providing a basis for adjusting treatment strategies^50,51^. In this way, the detection technology of CTCs can not only help us understand the dynamic changes of the tumor microenvironment, but can also be combined with the study of TLS to provide a dual perspective for developing new treatments and improving tumor management.

Certainly, the functions of the TLS-associated genes we identified in TLS have not been fully elucidated. Several studies have demonstrated the use of deep learning and graph network analysis techniques to predict potential biological associations in different biomedical domains (metabolite-disease associations, drug-induced liver injury, non-coding RNA–protein interactions). These approaches may provide a framework for parsing complex interactions between TLS genes and colorectal cancer^52–55^. The complex network of interactions between LS-associated genes and other genes or proteins is learned through a number of newly discovered bioinformatics algorithms to reveal their mechanisms of action in the tumor microenvironment. Such techniques can be applied to extract features from large-scale gene expression data to help identify TLS gene expression patterns that are closely associated with colorectal cancer prognosis^56–60^.

In summary, based on bioinformatics analysis, this study screened the TLS-related genes in the TCGA-COAD set to construct a 7-gene prognostic model, and verified the effectiveness of the model in evaluating the prognosis survival rate, immune characteristic differences and genomic mutation differences of patients. At the same time, the potential small molecule drugs acting on COAD were explored to provide guidance for clinical targeted medication of COAD. Of course, there are some limitations in this study. For example, the research data are derived from open databases, and there are some systematic errors. In addition, the model feature genes mined in this study need to be further verified by cell or tissue experiments to further verify their actual role in cancer progression or patient prognosis.

## Data Availability

The datasets generated and/or analyzed during the current study are available in the [GSE17536] repository, [https://www.ncbi.nlm.nih.gov/geo/query/acc.cgi?acc = GSE17536].

## Abbreviations

COAD: Colon adenocarcinoma
TLS: Tertiary lymphoid structure
TCGA: The cancer genome atlas
TMB: Tumor mutation burden
TME: Tumor microenvironment
DEGs: Differentially expressed genes
OS: Overall survival
GDSC: Cancer drug sensitivity genomics
IC50: The maximum half-inhibitory concentration
